# Effect of *Urtica dioica* Leaf Alcoholic and Aqueous Extracts on the Number and the Diameter of the Islets in Diabetic Rats

**Published:** 2013

**Authors:** Durdi Qujeq, Mohsen Tatar, Farideh Feizi, Hadi Parsian, Alieh Sohan Faraji, Sohrab Halalkhor

**Affiliations:** 1 *Cellular and Molecular Biology Research Center (CMBRC), Babol University of Medical Sciences, Babol, Iran.*; 2 *Department of Biochemistry and Biophysics, Faculty of Medicine, Babol University of Medical Sciences, Babol, Iran.*; 3 *Department of Anatomical Sciences, Faculty of Medicine, Babol University of Medical Sciences, Babol, Iran.*

**Keywords:** Diabetes, pancreas, *Urtica dioica*, rat, alcoholic and aqueous extract

## Abstract

*Urtica dioica *has been known as a plant that decreases blood glucose. Despite the importance of this plant in herbal medicine, relatively little research has been down on effects of this plant on islets yet. The objective of the current study was to evaluate the effect of dried *Urtica dioica *leaf alcoholic and aqueous extracts on the number and the diameter of the islets and histological parameters in streptozocin-induced diabetic rats. Six rats were used in each group. Group I: Normal rats were administered saline daily for 8 weeks. Group II: Diabetic rats were administered streptozotocin, 50 mg/kg of body weight; Group III: Diabetic rats were administered dried *Urtica dioica *leaf aqueous extracts for 8 weeks; Group IV: Diabetic rats were administered dried* Urtica dioica *leaf alcoholic extracts for 8 weeks. The animals, groups of diabetic and normal, were sacrificed by ether anaesthesia. Whole pancreas was dissected. The tissue samples were formalin fixed and paraffin embedded for microscopic examination. Histologic examination and grading were carried out on hematoxylin-eosin stained sections. The effects of administration of dried* Urtica dioica *leaf alcoholic and aqueous extracts to diabetic rats were determined by histopathologic examination. The pancreas from control rats showed normal pancreatic islets histoarchitecture. Our results also, indicate that the pancreas from diabetic rats show injury of pancreas tissue while the pancreas from diabetic rats treated with dried* Urtica dioica *leaf alcoholic and aqueous extracts show slight to moderate rearrangement of islets. According to our findings, dried* Urtica dioica *leaf alcoholic and aqueous extracts can cause a suitable repair of pancreatic tissue in streptozocin-induced diabetic experimental model.

*Urtica* *dioica *has been known as a medicinal plant in the world (1). It is known for its use against diabetes in folk medicine (2). Several experimental studies have investigated the role of *Urtica dioica *(UD) as a hypoglycemic agent (3-7). It has been widely used in traditional natural treatments for diabetes (1, 5) and based on these data has been applied to medical use for many years in the treatment of diabetic diseases (6). UD is also known for its use against diabetes in folk medicine (7-9). As reported by many other investigators *U. dioica *is a plant belonging to the plant family Urticaceae. The blood sugar lowering effect of *U. dioica *as a medicinal plant has been reported (10).

*U.* *dioica *leaves extract given parenterally possesses a hypoglycemic effect in alloxan hyperglycemic rats (11). *Urtica dioica *extract had hypoglycemic and antidiabetic activities (12). The extract of UD was unable to enhance the glucose utilization directly (13).

In view of the above mentioned varied unique pharmaceutical and biological application of dried *Urtica dioica *leaf extracts**, **it may be pragmatic to say that this plant have the potential to be used as active compound in diabetes. The objective of the current study was to evaluate the effect of dried* Urtica dioica *leaf alcoholic and aqueous extracts on the number and the diameter of the islets and histological parameters in streptozocin-induced diabetic rats.

## Materials and methods

**Preparation of** ***Urtica*** ***dioica*** **leaves**

*Urtica* *dioica *leaves were purchased from herbalists in Babol, Iran. The leaves were separated and dried at room temperature while keeping away from direct sunlight and then were grounded into powder.

**Preparation of the aqueous** **and** **alcoholic** **powder of *Urtica dioica*** **leaf extracts**

In order to prepare the aqueous concentrate, 200 g of room temperature dried *Urtica dioica *leaves were infused in two litres of distilled water and were heated at 80 ºC (Behdad–water bath) for 30 minutes with occasional stirrings. The extract of *Urtica dioica *leaves was filtered. The non soluble part was then separated using a mesh and the solution was passed two times through Whatman paper No. 2, and the aqueous part was evaporated on a rotator evaporator (K-1Karwerke, GMBH SCo KG, Germany, TYP: RVo6-ML, 010388949) at 75°C to reduce the solution volume to 1/5 of the initial value. For the preparation of ethanolic solution, 200 g of dried leaves was dissolved in one litre of 96% ethanol and mixed by a shaker (Labtron–Ls-100) for 24 hrs at room temperature. Again the non soluble part was separated using a mesh and the solution was passed two times through Whatman paper No. 2. The macerate was filtered, and ethanol was evaporated on a rotator evaporator at 60°C under the vacuum to reduce the solution volume to 1/5 of the initial value. The powder of *Urtica dioica *leaves was percolated by aqueous (60º) solvent for 24 hrs. The extract was filtered and concentrated under vaccum at 60ºC. Chromatography and purity tests for qualification analysis were carried out. Both aqueous and ethanolic solutions were kept four days under hood to let the solvents evaporate. Finally, the extract was stored at -20 ºC until its use within 1 week.

**Isolation and purification of powder of** ***Urtica dioica*** **leaf extracts by chromatography**

1 ml of crude extract was loaded onto a sephadex G-20 packed column and the column was washed with 10 mM Tris-HCl, pH 7.2. Fractions with different optical densities were collected. All fractions were stored at−20°C. The fraction 2 was the most effective at increasing glucose uptake *in vitro *as well as *in vivo *in hyperglycemic rats. 

**Animals**

Male adult rats of 200-250 g were fed on pellet diet and tap water for full acclimatization. The rats were divided into four groups (each group including 6 rats). Rats of closed colony were prepared from the animal center of Babol University of Medical Sciences, Babol, IRAN. Age-matched rats were used as control animals. The rats were housed in suspended bracket cages in a climate-controlled room at 22±5 ºC with 12L:12D lighting cycle. Feed and water were provided *ad libitum*. All procedures were in accordance with animal experimental guidelines of Babol University of Medical Sciences. The approval of the Ethics Committee of Babol University was also obtained (# 3546, 1390/2/7; 1815, 1389/7/6).

**Diabetic induction**

Diabetes was induced by intraperitoneal injection (50 mg/kg, i.p) of 0.6 ml of freshly prepared solution of streptozotocin (STZ) dissolved in distilled cold normal saline. Rats were not fed 14 hrs before injection. In addition, 0.7 ml normal saline was injected to rats in the control groups. Rats with fasting glycemia more than 250 mg/dl were used as diabetic. Blood samples for glucose measurements were taken from the tail vein. Diabetes was confirmed by measuring the glucose concentration.

**Animals administration**

Animals were randomly divided into four groups. Six rats were used in each group: (I) the normal group, normal saline daily administered for 7 days. (II) the diabetic group, animals that received 50 mg/kg streptozotocin intraperitoneally and did not receive any extract. (III) the treatment group, diabetic induced by one dose of intraperitoneally injection of 50 mg STZ which then received 15 mg/kg daily aqueous extract of *Urtica dioica *leaf*, *for 56 days. (IV) the hyperglycemic animals that received 50 mg/kg streptozotocin intraperitoneally and treated with ethanolic extract. Before experiments and after 72 hrs, while rats were not fed for 14 hrs, their blood samples were obtained from tail vein. Glucose and insulin of blood serum were measured. Then, 24 hrs after obtaining blood samples rats were treated orally with 10 mg/kg/day *Urtica dioica *leaf extracts or distilled water (as the control) for 56 days. Upon completion of this period, the blood samples were obtained again to measure the glucose and insulin levels. 

**Histological tests**

Eight weeks from the start of the experiment, all animals in the four groups were sacrificed by ether anesthesia. Whole pancreas was dissected. The tissue samples were fixed in formalin %10 and paraffin embedded for micros-copic examination in accordance with routine laboratory procedures. Histopathology examination and grading were carried out on haematoxilin and eosin stained sections at 5-μm thickness (Leitz 1512, Germany). The size and cellularity of islets were evaluated by olympus BX41 light microscope and motic image plus 2.0 ML.

**Statistical analysis**

All values have been presented as mean ± standard error. All tests were carried out in triplicate. Statistical analysis were done using SPSS version 16.0. The significance of differences between the mean values were determined by analysis of variance (ANOVA) or was determined using an unpaired two-tailed Student’s t-test, and a *p *value of less than 0.05 was considered statistically significant.

## Results

**Fig 1:**
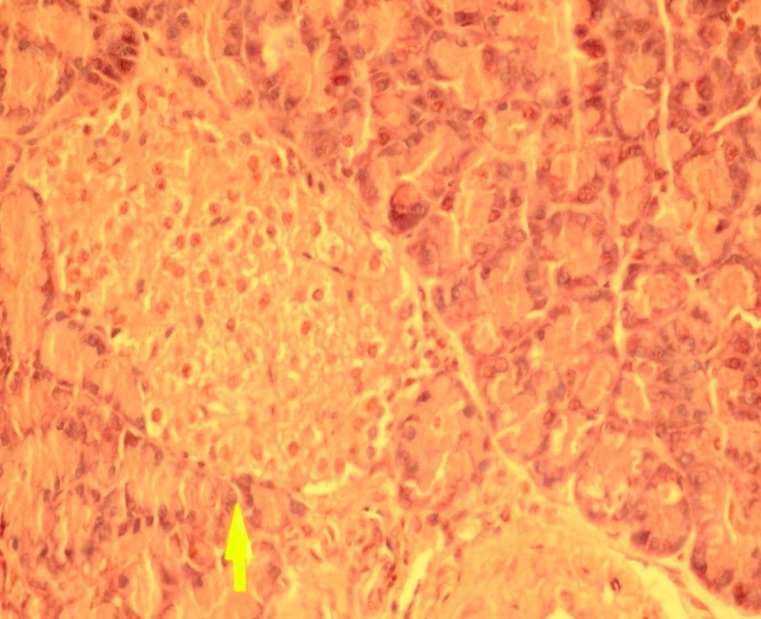
The pancreas from control rat (haematoxylin and eosin, original magnification ×40).

**Fig 2:**
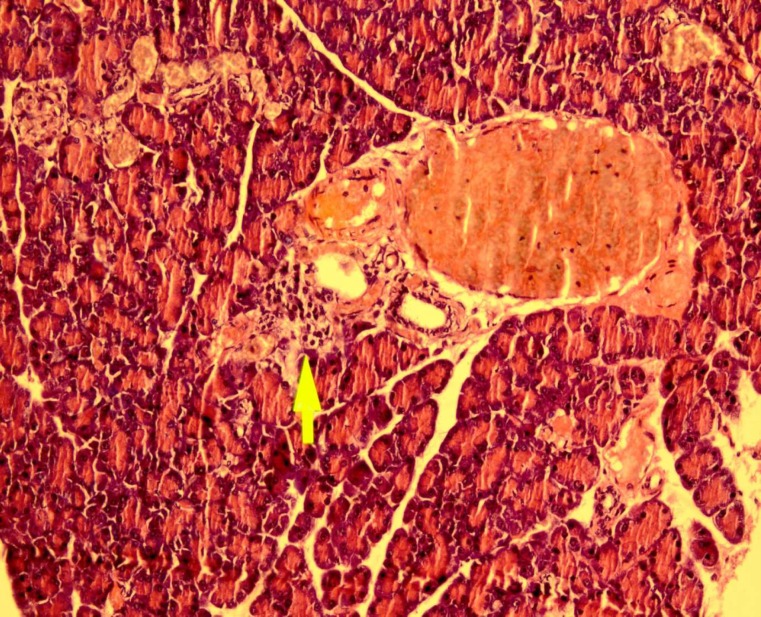
The pancreas from didbetic (haematoxylin and eosin, original magnification ×40).

**Fig 3:**
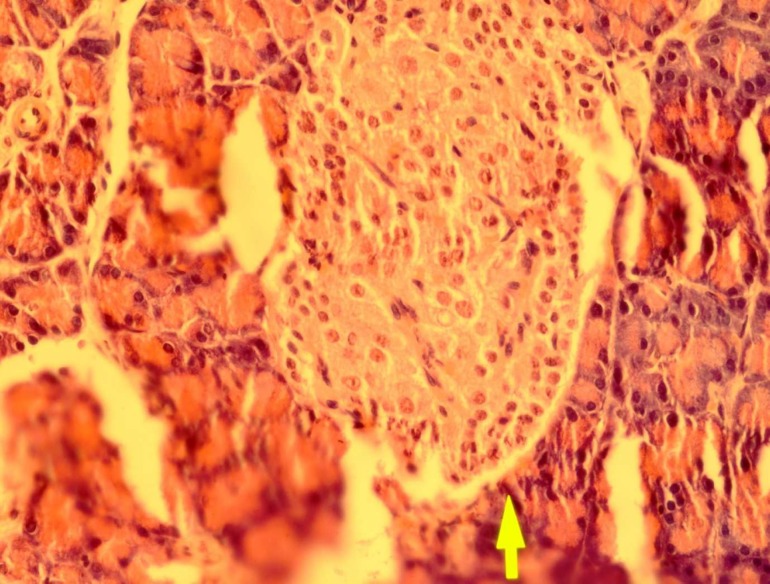
The pancreas from a diabetic rat treated with ethanolic powder of *Urtica Dioica *leaf extracts (haematoxylin and eosin, original magnification ×40).

**Fig 4:**
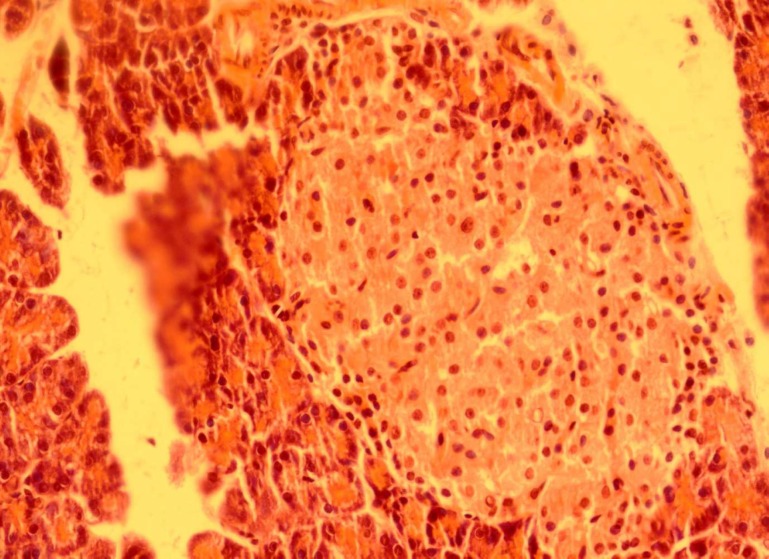
The pancreas from a diabetic rat treated with aqueous powder of *Urtica Dioica *leaf extracts ( haematoxylin and eosin, original magnification ×40).

Effect of dried* Urtica dioica *leaf alcoholic and aqueous extracts on number of islets and islets diameter in normal and diabetic rats was shown in Table 1. Our results indicate that administration of streptozotocin intraperitoneally reduce the cellula-rity and diameter of islets compared to the control group. Administration of dried* Urtica dioica *leaf ethanolic extracts significantly increased the cellularity and diameter of islets in treated group compared to diabetic group. Also, the diameter and cellularity of islets in rats treated with* Urtica dioica *leaf aqueous extracts significantly increased. There were no abnormalities in the pancreas of normal rats (Fig.1). Diabetic rats showed injury of pancreas tissue (Fig.2). The Diabetic rats treated with ethanolic *Urtica dioica *leaf extracts showed moderate repair of pancreatic tissue (Fig.3). Diabetic rats treated with aqueous *Urtica dioica *leaf extracts also showed slight to moderate repair of pancreatic tissue, probably due to partial 

regeneration of the pancreatic cells (Fig.4).

**Table 1:** Comparison of the effect of *Urtica dioica *leaf extracts on the islets diameter in normal and diabetic rats

**Group treatment **	**Islets diameter(µm)**
Normal	34.94± 24.60
Diabetic	13.20±7.35
Diabetic +* aqueous Urtica dioica* leaf extracts	25.58±12.18
Diabetic +* alcoholic Urtica dioica *leaf extracts	26.37±14.67

All data have been presented as mean ± standard error, P<0.05

## Discussion

Histopathological aspects of pancreatic structure in diabetic models have received less attention. In the present study, we assessed the pancreatic changes at light microscopy level and studied the effect of dried *Urtica dioica *leaf alcoholic and aqueous extracts on the pancreas of diabetic rats. According to our findings, dried *Urtica dioica *leaf alcoholic and aqueous extracts may have been a protective effect or repairative effect on iletes of pancreas tissue in experimental model. The cellularity and diameter of islets were decreased after streptozocin treatment. This study showed that the administration of dried *Urtica dioica *leaf extracts after induction of diabetes in rats protects the islets so diameter and cellularity of islets were not diminished or may be replaced by duplication of remained cells or pancreatic ducts. (4 ). These data confirm earlier studies (5, 6).

Moreover; investigators showed that *Urtica dioica *was a potent stimulator of insulin release of β-cells (6). Also others reported that *Urtica dioica *has hypoglycemic activity and β-cell regenerative potency (14). But, our observations are not in agreement with the results obtained by other investigators (15) who did not observe a hypoglycemia activity of aqueous extract of the powder of *Urtica dioica *leaf extracts. In this study, in treatment group the dried *Urtica dioica *leaf extracts can cause repair activity after eight weeks of administration in streptozotocin diabetic rats. Therefore, administration of dried* Urtica dioica *leaf extracts after inducing diabees in rats could prevent the pancreas damage. The exact mechanism of the pancreatic changes due to dried* Urtica dioica *leaf extracts in animal diabetic models is not clear, but there are several possible mechanisms of these alterations in the pancreatic cells. These changes may be influenced by components present in the dried *Urtica dioica *leaf extracts. Based on the beneficial effects of plant materials in improving tissue regeneration, we hypothesized that effect of dried* Urtica dioica *leaf extracts may promote the pancreatic ability to normal function. 

The improved function of pancreas after treatment could be attributed to this beneficial dried* Urtica dioica *leaf extracts effect. An accelerated recovery and improved function after pancreatic injury could be attributed to the promotion of endogenous repair pathway. Therefore, in future it will be important to examine the effect of dried* Urtica dioica *leaf extracts in diabetic experimental model. 
